# Pancreatic Stromal Tumor of Nerve Sheath Origin Treated by Pancreatoduodenectomy

**DOI:** 10.1155/1991/21629

**Published:** 1991

**Authors:** L. Pereira-Lima, A. N. Kalil, M. B. Furian

**Affiliations:** 1 Department of Surgery Fundaçáo Faculdade Federal de Ciêneias Médicas de Porto Alegre Santa Casa Hospital RS Porto Alegre Belize; 2 R. Tobias da Silva 66–2° a RS Porto Alegre 90.460 Belize

## Abstract

A pancreatic sarcoma of nerve sheath origin is reported in a 28-year-old female patient, who presented with melaena. Preoperative imaging showed an 8.5 cm diameter mass in the head of pancreas. There was bleeding from the papilla of Vater at endoscopy and a highly vascular lesion on arteriography. The patient was submitted to proximal pancreatoduodenectomy and remains symptom-free at 1 year follow-up.


*HPB Surgery*, 1991, Vol. 4, pp. 165–169
Reprints available directly from the publisher
Photocopying permitted by license only

© 1991 Harwood Academic Publishers GmbH
Printed in the United Kingdom

### CASE REPORT

### PANCREATIC STROMAL TUMOR OF NERVE SHEATH ORIGIN TREATED BY PANCREATODUODENECTOMY

L. PEREIRA-LIMA, A.N. KALIL and M.B. FURIAN
*Department of Surgery, Fundaçáo Faculdade Federal de Ciêneias Médicas de Porto Alegre, Santa Casa Hospital, Porto Alegre, RS, Brasil*

*(Received 3 December 1990)*

A pancreatic sarcoma of nerve sheath origin is reported in a 28-year-old female patient, who presented with melaena. Preoperative imaging showed an 8.5 cm diameter mass in the head of pancreas. There was bleeding from the papilla of Vater at endoscopy and a highly vascular lesion on arteriography. The patient was submitted to proximal pancreatoduodenectomy and remains symptom-free at 1 year follow-up.

KEY WORDS: Pancreatic stromal tumour, pancreatoduodenectomy

### INTRODUCTION

Pancreatic tumors of mesenchymal origin are extremely rare. Only a few cases have been reported, relating to different kinds of sarcoma, but full appraisal of the histological origin by immunohistochemical methods is scarce[1-3]. We therefore report a case of a pancreatic stromal tumour with nerve sheath differentiation.

### CASE REPORT

The patient was a 29 year-old white female, whose main complaint was of tarry stools and anaemia. Upper gastrointestinal endoscopy revealed bleeding from the papilla of Vater. Ultrasonography showed a mass in the head of the pancreas, 8.5 cm in maximal diameter, of mixed echogenicity and compressing the inferior vena cava. Abdominal CT scan confirmed these findings (Figure 1). There was no dilatation of the biliary tract. Selective arteriography of the coeliac trunk and superior mesenteric artery identified a hypervascular lesion in the pancreatic region, fed mainly from the inferior pancreaticoduodenal artery (Figure 2).

At laparotomy there was a large mass in the head of the pancreas with a rich collateral circulation, but the tumour was still mobile. There was no evidence of dissemination. The bile duct appeared normal, as did the body and tail of pancreas. Proximal pancreatoduodenectomy was performed including partial gastrectomy.

---

Address correspondence to: Professor Luiz Pereira-Lima, R. Tobias da Silva, 66–2° a, 90.460 - Porto Alegre - RS, Brasil

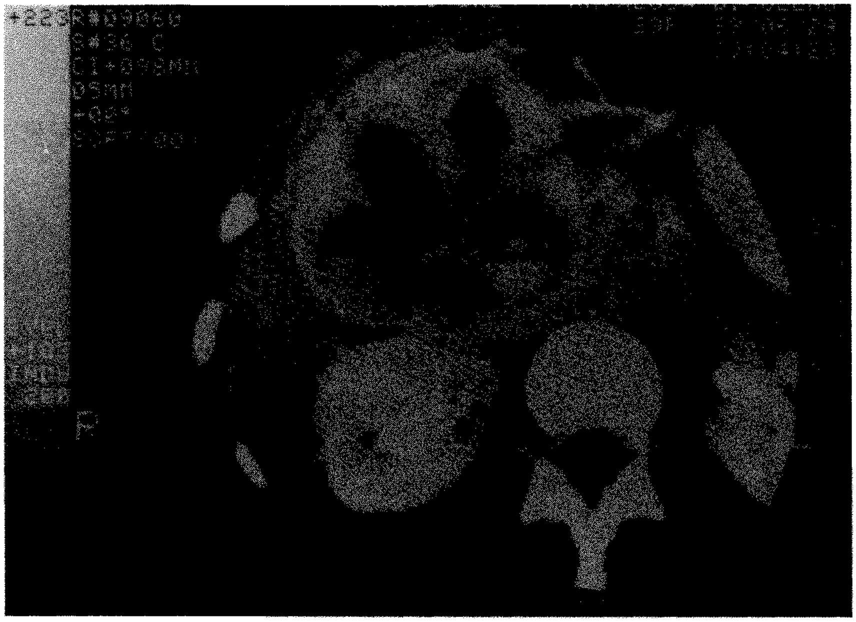

**Figure 1** A mass in the head of pancreas on CT scan.

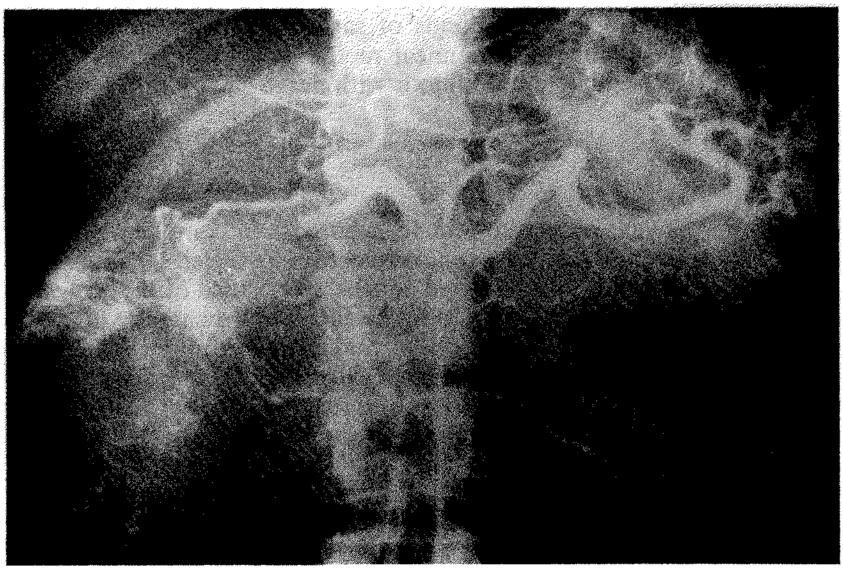

**Figure 2** Selective arteriography showing hypervascular tumour in the region of the head of pancreas.

Macroscopic examination of the operative specimen (Figure 3) showed a tumour of elastic consistency with several thin-walled loculi containing haemorrhagic material. One of these cavities opened into the duct of Wirsung. Microscopic examination revealed a malignant neoplasm with a predominance of spindle cells. There were no metastatic deposits among the 20 lymph nodes examined.

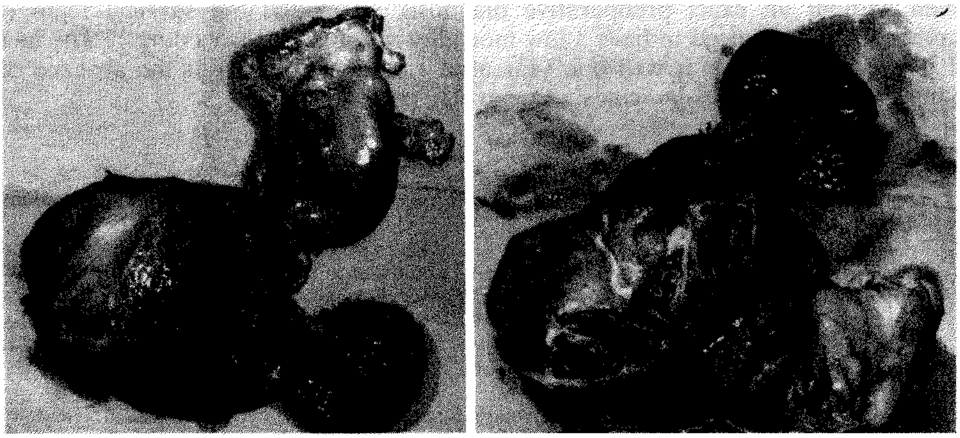

**Figure 3** Cut pancreatoduodenectomy specimen showing a tumour with cystic haemorrhagic cavities.

To determine the origin of the tumour, an immunohistochemical study was performed. Deparaffinized sections were incubated with the following panel of monoclonal and/or polyclonal antibodies, localization was by means of the avidin biotin (ABC) immunoperoxidase method. The results were negative for low molecular weight cytokeratin (35BH11), high molecular weight cytokeratin (34BE12), epithelial membrane antigen, carcinoembryonic antigen, muscle actins (HHF35), desmin (Clone 33), melanoma specific antigen (HMB45), CD57(Leu-7), type IV collagen, nerve growth factor receptor protein (NGFR 5), neurofilament (2F11,NRV170), myelin basic protein, CD45 (T200) and synaptophysin. The results were uniformly positive for vimentin (Dako) and variably positive for S100 protein. The final diagnosis was consistent with a stromal tumour of nerve sheath origin.

The postoperative course was uneventful. At the time of writing, one year later, the patient remains asymptomatic with no evidence of recurrence.

### DISCUSSION

In a review of more recent literature on pancreatic tumours, Kloppel[3] records cases of lymphangioma, leiomyosarcoma, haemangioendothelioma, rhabdomyosarcoma, myeloma, malignant fibrous histiocytoma and haemangiopericytoma. In this case conventional hystological studies only could identify a spindle-cell sarcoma. The evidence that we were dealing with a stromal tumor of nerve sheath origin came later through immunochemical studies using several different antibodies.

It is important to note that the prognosis of such lesions, when they are removed, is much better than it is for tumours of epithelial origin. They are slow-growing even if they show aggressive histological features. Characteristically they are hypervascular and have a false capsule, which can make surgical removal difficult. Nevertheless, resection is well worthwhile because of the favourable prognosis (compared with epithelial tumours of the pancreas).

The Whipple operation is the procedure of choice for a periampullary tumour, even without an exact preoperative diagnosis[4], provided the surgical team is sufficiently experienced to have a low mortality rate for this procedure[1,5]. The fact that our patient remains well one year after radical excision plus the absence of lymph node metastases indicates a favourable prognosis.

### *References*

1. Garvey, J.F.W., Ng, A., England, J.F. and Sheldon, D.M. (1989) Malignant fibrous histiocytoma of the pancreas. *HPB Surgery*, **1**, 233–237
2. Ishiguchi, T., Shimamoto, K., Kaii, O., Sakai, M., Ishigaki, T., Sakuma, S., Hasegawa, H. and Nimura, Y. (1986) Malignant fibrous histiocytoma of the pancreas. *Rinsho Hoshasen*, **31**, 655–658
3. Kloppel, G. (1989) Cancer of the Pancreas. In *Cancer of the Bile Ducts and Pancreas*. Preece, P.E., Cuschieri, A., Rosin, R.D. (eds) pp. 113–138. Philadelphia: W.B. Saunders Company
4. Moossa, A.R. (1989) Surgical treatment of pancreatic cancer. In *Cancer of the Bile Ducts and Pancreas*. Preece, P.E., Cuschieri, A., Rosin, R.D. (eds) pp. 197–208. Philadelphia: W.B. Saunders Company
5. Brooke, W.S., Maxwell, J.G. (1966) Primary sarcoma of the pancreas. *American Journal of Surgery*, **112**, 657–661

(*Accepted by S. Bengmark on 3 December 1990*)

### INVITED COMMENTARY

Malignant spindle-cell tumours of the pancreas are distinctly uncommon. In the past their histogenesis has been disputed, some appearing to be epithelial in origin, others mesenchymal. Electron microscopy and immunohistochemistry now make it possible to classify spindle-cell neoplasms into those of epithelial, mesenchymal, nervous or lymphoid tissue origin.

Although Professor Pereira-Lima and his colleagues call this a case of "pancreatic stromal tumour with nerve sheath differentiation", a less confusing term might be "malignant schwannoma of the pancreas". Malignant schwannomas are often multiple, affecting the limbs of patients with neurofibromatosis[1], but they have been described in the stomach and duodenum and twice before in the pancreas[2,3].

In the present case, positive categorisation as a mesenchymal tumour of nerve sheath origin rests on the presence of spindle cells, uniformly positive staining for vimentin (a mesenchymal marker) and variably positive staining for S100 protein (a nerve sheath marker). Markers were negative for epithelial cells, smooth muscle, striated muscle and nerve cells, together with melanoma specific antigen. The authors have provided no details of the tissue preparation for immunohistochemistry, yet the method and duration of fixation can affect interpretation of the results. For example, desmin and neurofilaments may not be labelled by monoclonal antibodies after 24 hours of formaldehyde fixation, and trypsinisation impairs the staining for cytokeratins[4]. Moreover, vimentin is positive not only in mesenchymal tumours but also in papillary-cystic neoplasm (solid and cystic tumour) of the pancreas[5] and in nearly all amelanotic melanomas[6]. Likewise, S100 protein is positive in all amelanotic melanomas[6] and one third of papillary-cystic neoplasms[5].

The lack of electronmicroscopic examination of this tumour does weaken the diagnosis of malignant schwannoma, since light-microscopic criteria can be inadequate[7]. Characteristic ultrastructural features of malignant schwannoma include the demonstration of basement membranes, functional complexes and interdigitating cytoplasmic extensions containing dense neurosecretory-like granules and axon-like structures[3,7].

The cystic centre of the tumour on scanning, its marked vascularity and — to a lesser extent — the young age of the patient made "ordinary" ductal carcinoma of the pancreas unlikely in this case. The preoperative differential diagnosis might therefore have included non-functioning endocrine tumour (which can be cystic), cystadenocarcinoma and papillary-cystic neoplasm, although the subsequent pathological findings helped to exclude these alternatives. The cystic nature and extreme vascularity of pancreatic schwannoma were features of the two previous case reports, one of which presented as a pulsatile mass and was initially drained into the stomach as an apparent pseudocyst[2]. This experience reinforces the need to biopsy the wall of all "pseudocysts"[8]. We agree with the authors that resectable tumours of the pancreas should be resected even without a precise preoperative diagnosis and that pancreatoduodenectomy is indicated for lesions in the head of the gland.

*References*

1. Ghosh, B.C., Ghosh, L., Huvos, A.G. and Fortner, J.G. (1973) Malignant schwannoma. A clinicopathologic study. *Cancer*, **31**, 184–190
2. Petersen, V.M., Hede, A. and Graem, N. (1982) A solitary malignant schwannoma mimicking a pancreatic pseudocyst. *Acta. Chir. Scand.*, **148**, 697–698
3. Eggermont, A., Vuzevski, V., Huisman, M., De Jong, K. and Jeekel, J. (1987) Solitary malignant schwannoma of the pancreas: report of a case and ultrastructural examination. *J. Surg. Oncol.*, **36**, 21–25
4. S-Y Leong, A. and Gilham, P.N. (1989) The effects of progressive formaldehyde fixation on the preservation of tissue antigens. *Pathology*, **21**, 266–268
5. Yamaguchi, K., Miyagahara, T., Tsuneyoshi, M., Enjoji, M., Horie, A., Nakayama, I., Tsuda, N., Fujii, H. and Takahara, O. (1989) Papillary cystic tumour of the pancreas: an immunohistochemical and ultrastructural study of 14 patients. *Jpn. J. Clin. Oncol.*, **19**(2), 102–111
6. Miettinen, M. and Franssila, K. (1989) Immunohistochemical spectrum of malignant melanoma. *Lab. Invest.*, **61**, 623–627
7. Chen, K.T., Latorraca, R., Fabich, D., Padgug, A., Hafez, G.R. and Gilbert, E.F, (1980) Malignant schwannoma. A light microscopic and ultrastructural study. *Cancer*, **45**, 1585–1593
8. Gillatt, D.A., Cooper, M.J. and Williamson, R.C.N. (1986) Pseudopseudocysts of the pancreas. *Pancreas*, **1**, 460–463

P. Watanapa
R.C.N. Williamson
Department of Surgery
Royal Postgraduate Medical School
Hammersmith Hospital
London W12 0NN, UK

